# Gender differences in the intention to get vaccinated against COVID-19: a systematic review and meta-analysis

**DOI:** 10.1007/s10389-021-01677-w

**Published:** 2022-01-07

**Authors:** Stephanie Zintel, Charlotte Flock, Anna Lisa Arbogast, Alice Forster, Christian von Wagner, Monika Sieverding

**Affiliations:** 1 Department of Psychology, Ruprecht Karls University Heidelberg, Hauptstr. 47-51, 69117 Heidelberg, Germany; 2 Research Department of Behavioural Science and Health, University College London, 1-19 Torrington Place, London, WC1E 6BT UK

**Keywords:** Covid-19, Vaccination intention, Gender differences, Health care workers, Systematic review, Meta-analysis

## Abstract

**Aim:**

We conducted a systematic review and meta-analysis to analyse gender differences in COVID-19 vaccination intentions.

**Subject and methods:**

PubMed, Web of Science and PsycInfo were searched (November 2020 to January 2021) for studies reporting absolute frequencies of COVID-19 vaccination intentions by gender. Averaged odds ratios comparing vaccination intentions among men and women were computed. Descriptive analyses of the studies were reported.

**Results:**

Sixty studies were included in the review and data from 46 studies (*n* = 141,550) were available for meta-analysis. A majority (58%) of papers reported men to have higher intentions to get vaccinated against COVID-19. Meta-analytic calculations showed that significantly fewer women stated that they would get vaccinated than men, OR 1.41 (95% CI 1.28 to 1.55). This effect was evident in several countries, and the difference was bigger in samples of health care workers than in unspecified general population samples.

**Conclusion:**

This systematic review and meta-analysis found lower vaccination intentions among women than men. This difference is discussed in the light of recent data on actual vaccination rates in different countries.

**Supplementary Information:**

The online version contains supplementary material available at 10.1007/s10389-021-01677-w.

## Introduction

The COVID-19 pandemic is among the greatest challenges of today’s time. As of mid October 2021, more than 236 million people have already been infected with SARS-CoV-2 with 4.8 million deaths worldwide (WHO 2021). Several vaccines have been approved (Zimmer et al. 2021) and after initial supply shortfalls, vaccination rates accelerated as of spring 2021. In high-income countries, vaccination rates with at least one dose varied between 60% and 80% by mid-October 2021 (Ritchie et al. 2021). In developing countries where supply with vaccines is scarce, vaccination rates are much lower, for example 52% in India and under 10% in most parts of Africa (Ritchie et al. 2021). It has been estimated that considering now dominating vaccines, vaccination rates of 85–95% are necessary to protect from a severe increase of infections (Weber et al. 2021).

Next to availability of vaccines, the intention to get vaccinated against COVID-19 is regarded as a key variable for predicting actual vaccination uptake; it has been shown in a meta-analysis that health-related intentions are causally linked to the respective health-related behaviours (Webb and Sheeran 2006). A high availability of vaccination doses is a necessary but not sufficient prerequisite of actual vaccination uptake. If intentions are too low in the general population or in specific subgroups, the success of a COVID-19 vaccination campaign is seriously threatened.

In this paper, we are interested in COVID-19 vaccination intentions as a function of gender. Research on other vaccines showed gender differences in vaccination status and intentions favouring men (Bish et al. 2011) which may transfer to the COVID-19 vaccine. Some early surveys also reported lower COVID-19 vaccination intentions among women (Galanis et al. 2020; Lin et al. 2021; Robinson et al. 2020). Lower vaccination intentions among women could be problematic for various reasons. Next to exposing themselves to the danger of a COVID-19 infection, women have a central role in ensuring the health of their children. Additionally, women are more likely to be health and social care workers who are at high risk of contracting and passing on COVID-19.

Before vaccines against COVID-19 were approved and vaccination programs started, worldwide surveys were undertaken to assess individuals’ intentions to get vaccinated against COVID-19 in the general population and among samples of health care workers (HCWs). The main goal of our study was to review and analyse the results of these surveys, investigating whether there are systematic gender differences in the intention to get vaccinated against COVID-19.

## Method

### Search strategy

The initial search was conducted on 19.11.2020 in PubMed, Web of Science and PsycInfo. We used the search terms (vaccination OR vaccine OR vaccinated) AND (corona OR coronavirus OR SARS-CoV-2 OR COVID-19) in combination with ‘refusal’, ‘hesitancy’, ‘hesitance’, ‘hesitation’, ‘acceptance’, ‘willingness’, ‘motivation’, ‘confidence’, ‘uptake’, ‘intention’, ‘attitude’, ‘emotion’, ‘opinion’, ‘trust’, ‘doubts’, ‘cognition’, ‘rejection’, ‘disapproval’, ‘belief’. This search identified 649 articles on PubMed, 192 on Web of Science and 17 on PsycInfo. We filtered results for the year 2020 to 2021 (because the global COVID-19 outbreak happened in 2020) and in PubMed for languages English or German. We used a method described by Bramer et al. (2016) to identify duplicates. The final number of articles for screening was 682.

**Fig. 1:**
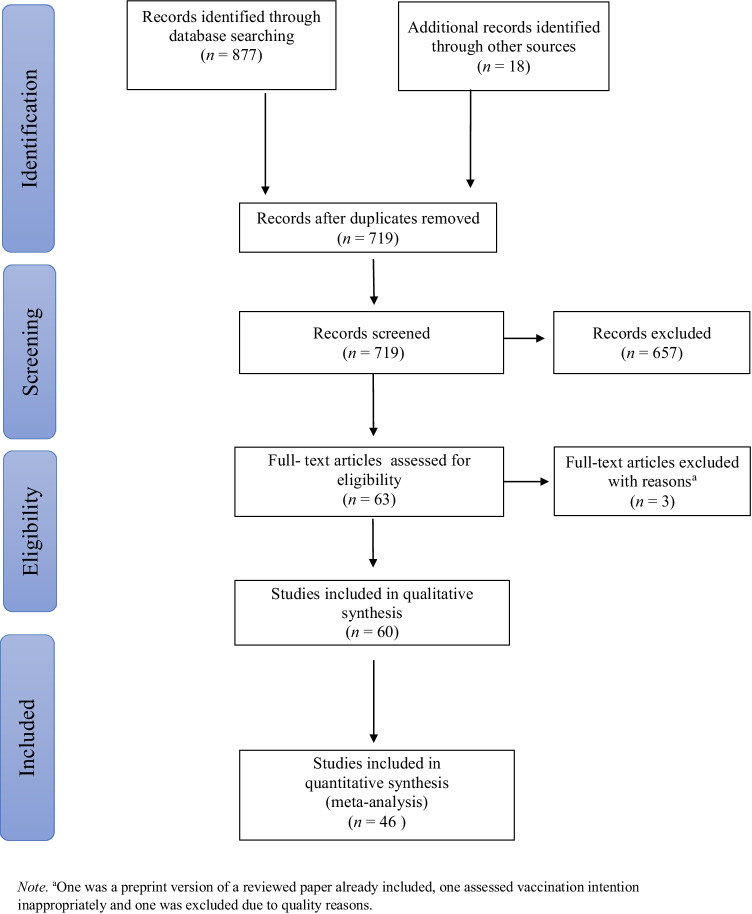
PRISMA flow diagram showing the study selection process

We identified 26 papers reporting gender specific data on vaccination intentions in any sort. We manually conducted a forward and backward citation search of those 26 papers. In this way, we identified a further 18 papers that reported gender data on vaccination intentions. Data extraction can be seen in Fig. 1.

After the initial search, we regularly checked data bases for new publications. Inclusions based on these searches can be seen in Fig. 1. The last search was conducted the 7th of January 2021 on all three databases. Subsequently we wrote to the authors (*N* = 50) who did not report the data needed for meta-analytic calculations in their articles. Lastly, we had to exclude 19 papers for various reasons.^1^

### Eligibility and exclusion criteria

The search results were screened for inclusion following these eligibility criteria: Primarily adult population, reported outcome: intention/willingness to get vaccinated against COVID-19 for men and women separately or gender differences statistically tested, available in English or German. Cross-sectional and longitudinal studies were included. Studies reporting an interventional/experimental design were excluded from our analysis (see Fig. 1 for study selection).

### Data analysis

Apart from looking at studies descriptively, we conducted meta-analytic calculations of averaged odds ratios. For this calculation, we first computed odds ratios using the exact frequency statistics reported in the papers or provided by the authors upon request. We made two different types of calculation: We compared ‘yes’-answers (including ‘definitely yes’- and ‘probably yes’-answers and similar answer options) with the remaining ‘non-yes’ answer categories which could also include ‘do not know’- or ‘not sure’-answers (in Table 1: YR, frequencies for yes vs. rest categories reported). We additionally compared ‘yes’-answers (including ‘definitely yes’- and ‘probably yes’-answers and similar answer options) with ‘no’-answers (including ‘definitely’ or ‘probably no’-answers and similar answer options, in Table 1: YN, frequencies for yes vs. no categories reported). For the meta-analytical calculations, we included 46 studies which provided the necessary data for the ‘yes’- versus ‘non-yes’ answers and used the metafor package in R described by Viechtbauer (2010) to compute mean gender effects of summarized odds ratios and confidence intervals. We also conducted a meta-analysis that was based on a smaller number of 40 studies which provided the necessary data for the ‘yes’- versus ‘no’ -answers. The results of this additional meta-analysis can be found in the Supplemental Material. We used a random effects model for our meta-analysis due to heterogeneity in our samples that (widely) differed in dimensions such as residence, age, and profession. As a result, we cannot assume that the effect estimates vary only because of chance differences from sampling participants, in which case a fixed-effect model is indicated (Riley et al. 2011). Restricted maximum likelihood estimation was used to fit a random-effects model to the data respectively as estimator to compute the heterogeneity τ^2^. In the meta-analysis, we used moderator analyses to determine if the effects of the studies differed depending on quality appraisal, month of assessment or being a healthcare worker or not (variable HCW). If recruitment took place over several months, the first month was coded. HCWs were chosen as a subgroup because it was the only group addressed by several studies. Knapp and Hartung adjustment was used to lower type I error rates (IntHout et al. 2014) and can be seen as a good replacement of the standard method (Jackson et al. 2017). Representativeness of samples was not used as a separate moderator because it was included in the quality rating. Owing to the rapidly evolving situation, it was not surprising that many papers were available as preprints which is unlikely to reflect the quality of research. For quality appraisal, we used the suitable aspects of already established tools as there were no comprehensive quality assessment tools that fitted for the survey studies. More detailed information can be found in the Supplementary Material. We adhered to PRISMA guidelines in the preparation and realization of our review.

**Table 1:** Description of studies (*n* = 60) included in review

Author	*N*, % female	Sample, Recruitment	Country^a^	Month	Publication Type	Intention Item	Quality Rating	Frequencies	Reported Gender Difference
Adebisi et al. (2020)	517, 43.1	Non-probabilty convenient sample, social media platforms	NGA	Aug	PP	‘Will you take COVID-19 vaccine?’ Yes/ No	8	YR YN	n.s. χ2 = 1.53 *P* value = 0.22
Akarsu et al. (2020)	759, 62.8	Snowball sample, social media platforms	TUR	Jun–Jul	PR	Exact wording n.a. Vaccination request: Yes, get vaccinated/ If it’s free, get it done/ No, do not get vaccinated/ Undecided	7	YR YN	Yes Women had more negative opinions (do not get vaccinated/ undecided) about getting vaccinated than men (*P* = 0.001).
Ali et al. (2020)	5,677, 69.5	Snowball sample, users of social media platforms	BHR, KWT, SAU, ARE, OMN, QAT […]^b^	Mar–Apr	PR	‘Suppose that a safe and effective coronavirus vaccine was available today. How likely are you to get yourself vaccinated?’ 1 (very likely) – 5 (very unlikely) 3 = neutral	7	YR YN	Yes (no exact statistics reported) Male subjects, reported a higher likelihood of vaccinating, expressed as ‘very likely’ and ‘somewhat likely’.
Al-Mohaithef and Padhi (2020)	992, 65.83	Snowball sample, social media platforms + e-mails	SAU	NA	PR	‘If vaccine against the coronavirus is available, I will take it’ Yes/No/Not Sure	3*	YR YN	n.s. *p* > 0.08
Barry et al. (2020)	1,512, 62.4	Convenient sample of health care workers, social media platforms + e-mail	KSA	Nov	PP	Exact wording n.a. Readiness to receive COVID-19 vaccine: as soon as possible/ waiting for a few months/ never accept a vaccine	7*	YR YN	Yes Males were 1.55 times more likely to accept a COVID-19 vaccine than females (*p* = .008).
Butter et al. (2020)	1,605, 68.6	Convenient sample of key workers and non-key workers, social media platforms, survey platform	GBR	Mar–Apr	PP	‘If a new vaccine was to be developed for coronavirus (COVID-19) and was available to you, would you accept it for yourself?’ Yes/ Do not know/ No	6*	NA	UNSURE Key worker sample: being female associated with vaccine hesitancy (OR = 1.96, [1.16–3.32]) not in the non-key worker sample (OR = 1.15, [0.83–1.59])
Callaghan et al. (2020)	5,009, NA	Quota sampling that mirrors population benchmarks, survey platform	USA	May–Jun	PP	‘Scientists around the world are working on developing a vaccine to protect individuals against the coronavirus. If a vaccine is developed, would you pursue getting vaccinated for the coronavirus?’ Yes/ No	8*^g^	YR YN	Yes Odds of vaccination refusal significantly higher for women (OR: 1.72, CI: 1.42, 2.08)
Daly and Robinson (2020)	7,547, 52.1	Nationally representative longitudinal study, Understanding America Study (UAS)	USA	Apr–Oct	PP	Exact wording n.a. How likely there were to get vaccinated for coronavirus when a vaccine becomes available to the public: Very likely/ Somewhat likely/ Undecided/ Somewhat unlikely/ Very unlikely (5 points)	11*	YR YN	Yes Females were at elevated relative risk of being undecided or unwilling to vaccinate (undecided: RRR = 1.41, 95% CI: 1.20–1.65; unwilling: RRR = 1.29, 21,995% CI: 1.14–1.46).
Davis et al. (2020)	1,008, 55.1	National household survey weighted to permit national estimates of parents with at least 1 child younger than age 18	USA	Jun	PP	‘If a vaccine against COVID-19 becomes available in the next 12 months, how likely are you to get it for [yourself/your child(ren)]?’ 1 (very likely) – 4 (somewhat likely)	8*	YR YN	UNSURE Sex was associated with parents’ likelihood to vaccinate their children and themselves in bivariate analyses *p* = .039. n.s. in multivariable analyses
Detoc et al. (2020)	3,259, 67.4	Adult general population & adult patients, social networks, e-mails, website of University Hospital, centers for COVID-19 diagnosis, medical centers	FRA	Mar	PP	Exact wording n.a. Willingness to get vaccinated: Strongly agree/ agree/ do not know/ disagree/ strongly disagree (5 points)	7*	YR	Yes Among men, more vaccine acceptors than among women, *p* < 0.005 In multivariable analyses, male gender remained associated with COVID-19 vaccine acceptance OR: 1.878 (1.529–2.306).
Dror er al. (2020)	1661, NA	Stratified for health care personnel at academic medical centers across Israels or members of general population, survey platform	ISR	Mar–Apr	PR	Exact wording n.a. Whether participants intend to accept future COVID-19 vaccination: Avoid/ Accept	6*^g^	NA	Yes Males more likely to accept the potential COVID-19 vaccine
Earnshaw et al. (2020)	845, 40.9	Invitation on crowdsourcing website	USA	Apr	PR	‘When a vaccine becomes available for the coronavirus, how likely are you to get it?’ Very likely/ Likely/ Somewhat likely/ Unlikely/ Not at all likely	9	NA	Yes Women were less likely to get a vaccine OR [ref. male]: 1.56 [1.02–2.39].
Echoru et al. (2020)	1,067, 26.8	Snowball sampling technique, social media + e-mails	West. UGA	Jul–Sept	PP	‘If the government of Uganda is to provide free COVID-19 vaccine, would you accept to be vaccinated?’ Yes/ No	6*	YR YN	Yes Male subjects were twice as likely to accept the vaccine (OR: 2.1; 95% CI: 1.56–2.71; *P* = 0.000).
Edwards et al. (2020)	2,717, 50.0*	Weighted to have a similar distribution to the Australian population, August ANUpoll	AUS	Aug	PP	‘The next questions ask about your views on a vaccine for COVID-19’ and then we ask ‘If a safe and effective vaccine for COVID-19 is developed, would you...’: Definitely not – Definitely (4 points)	10	YR YN	Yes Females were less likely than males to intend to get the vaccine, and more likely to be hesitant and resistant, *p*s < 0.05 (univariat).
Faasse and Newby (2020)	2,174, 75.2	Convenient sample, Facebook advertisement + post	AUS	Mar	PP	Exact wording n.a. How likely it is that participants would choose to have a vaccination for the COVID-19 coronavirus, if there was a safe and effective vaccine developed: Would definitely get the vaccine/ Would probably get the vaccine/ Unsure if I would get the vaccine or not Would probably not get the vaccine/ Would definitely not get the vaccine	7	NA	n.s. No demographic differences in vaccine intentions by gender (*p* = 0.429)
Fisher et al. (2020)	991, 51.5	Nationally representative sample, probability-based research panel AmeriSpeak	USA	Apr	PR	‘When a vaccine for the coronavirus becomes available, will you get vaccinated?’ Yes/ Not sure/ No	12	YR YN	Yes Participant characteristics associated with a higher chance of responding ‘no’ or ‘not sure’ versus ‘yes’ were being […] female. After adjustment for differences in participant characteristics: characteristics, such as female sex […] were associated with vaccination intent but did not consistently achieve statistical significance for both response categories.
Gadoth et al. (2020)	609, 68.8%	Health care workers employed by UCLA	USA	Sept–Oct	PP	Exact wording n.a. Prospective acceptance of a novel coronavirus vaccine	6	NA	n.s. *p* = 0.2643 OR [female]: 1.28, (0.83–1.98)
Grech et al. (2020a)	128, NA	General Practitioners & GP-trainees, e-mails from mailing list of Malta college family doctors	MLT	Sept	PR	‘Based on this information, how likely are you to take the COVID-19 vaccine?’ 1 (likely) – 5 (unlikely), with 3 = undecided	8^g^	YR YN	n.s. Males were more likely to take the vaccine than females (70% vs. 54%) but this was not statistically significant.
Grech and Gauci (2020)	852, NA	Students, academic & management/support staff at the University of Malta Faculties of Health Sciences, Dentistry & Medicine, e-mail via faculty secretaries	MLT	Sept	PR	See Grech, Bonnici & Zammit	8^g^	YR YN	Yes Males were likelier to take the COVID- 19 vaccine than females (70% vs 53% respectively, chi =25.7, *p* < 0.001).
Grech et al. (2020b)	1,002, NA	Malta’s government sector health care workers, e-mail	MLT	Sept	PR	See Grech, Bonnici & Zammit	8^g^	YR YN	Yes Males were likelier to take the vaccine than females (chi = 13.2, *p* = 0.0003).
Grüner and Krüger (2020)	2,077, NA	Health care studens, non-healthcare students, healthcare professionals	DEU	May–Aug	PR	Exact wording n.a. Willingness to be vaccinated against COVID-19	4^g^	YR YN	Yes Willingness to be vaccinated higher for men than for women *p* = 0.1
Guidry et al. (2021)	788, 50.0	Quotas for gender + race distribution, survey research firm	USA	Jul	PR	‘I intend to get the COVID-19 vaccine when it becomes available.’ Strongly agree – Strongly disagree (6 point)^c^	9	NA	Yes Men more likely to express intent to get a future COVID-19 vaccine (*p* = 0.003)
Hacquin et al. (2020)	4,027, 52.5	Representative sample of population, polling firm	FRA	May– Sept	PP	Exact wording n.a. Agree to get vaccinated if a vaccine against the COVID-19 is available: Certainly/ Probably/ Probably not/ Certainly not	11	YR YN	Yes Women (39.4%) were more likely than men (34.4%, p < 0.001) to refuse the vaccine OR: 0.63 [0.54–0.72]
Head et al. (2020)	3,159, 52.8	Market research firm using US-panel of people 18+ and able to understand English	USA	May	PR	‘How likely is it that you’ll get a COVID-19 vaccine, if it becomes available?’ Very unlikely/ Somewhat unlikely/ A little unlikely/ Neither likely nor unlikely/ A little likely / Somewhat likely /Very likely (7 point)	10	NA	Yes Sex significantly associated with intent to get vaccinated (*p* = .013, bivariate analysis), men show higher intent.
Khubchandani et al. (2021)	1,878, 52.0	Convenient sample, crowdsourcing website, social media and other networks	USA	Jun	PR	‘If a vaccine was available that would prevent coronavirus infection, how likely is it that you would get the vaccine/shot?’ Very likely/ Somewhat likely/ Not likely/ Definitely not	7*	YR YN	UNSURE Dichotomized Chi-squared test: n.s. *p* = 0.81 In multiple logistic regression, females had statistically significant higher odds of vaccine hesitancy (AOR: 1.44 [1.12–1.84]
Kose et al. (2020)	1,138, 72.5	Convenient sample of health care workers, social media	TUR	Sept	PR	‘If effective and safe vaccine is available for COVID-19, do you accept to be vaccinated with?’ Yes/ Indecisive/ No	4	YR YN	Yes Men were willing to get the vaccine. Gender associated with willingness to get vaccine, *p* = .001
Kwok et al. (2020)	1,205, 90.0	Convenient sample of nurses, online self-administered	HKG	Mar–Apr	PP	Exact wording n.a. How likely participants will take the COVID-19 vaccine when available: 0 (definitely no) – 10 (definitely yes)	6*	NA	n.s. OR (female): =.98 [0.68, 1.42]
La Vecchia et al. (2020)	1,055, 51.8	Nationally representative sample, market research company	ITA	Sept	PR	Exact wording n.a. Potential COVID-19 vaccine: (Probably) Yes/ (Probably) No (4 categories)	9	YR YN	Not reported
Lazarus et al. (2020)	13,426, 53.5	Representative sample, online, telephone + mail solicitation	BRA, CAN, CHN, ECU, FRA, […]^d^	Jun	PR	‘If a COVID-19 vaccine is proven safe and effective and is available to me, I will take it.’ Completely agree/ somewhat agree/ neutral,no opinion/ somewhat disagree/ completely disagree(5 points)	12	YR	Yes Gender differences were small, but the univariate association for both questions suggested that men were slightly less likely to respond positively than women, with an OR of 0.84 (95% CI (0.78, 0.91)) of men responding positively relative to women for the general question.
Lin et al. (2020)	3,541, 51.9	Non-representative sample, social media platform	CHN	May	PR	‘If a vaccine against COVID-19 was available on the market, would you take it?’ Definitely not/ Probably not/ Probably yes/ Definitely yes (4 point)	9	YR YN	n.s. *p* = 0.137 in univariable analysis
Loomba et al. (2020)	4,001, 57.0 GBR 4,000, 55.0 USA	Nationally representative sample, online panel	GBR USA	Sept	PP	‘If a new coronavirus (COVID-19) vaccine became available, would you accept the vaccine for yourself?’ Yes, definitely/ Unsure but leaning towards yes/ Unsure but leaning towards no/ No definitely not (4 point)	9	YR YN	Yes In both countries, females are more likely than males to refuse a COVID-19 vaccine, with a larger effect-size in the US (odds ratio 2.02, 95% percentage interval (PI): 1.78 to 2.29) than the UK (OR 1.44 [1.25, 1.63]).
Lucia et al. (2020)	168, 57.0	Medical students from one allopathic medical school in Southeast Michigan	USA	NA	PR	Exact wording n.a. COVID-19 vaccine uptake: Vaccine Acceptance group/ Vaccine hesitant group	5	YR YN	n.s. Demographic variables were not predictive of COVID-19 vaccine uptake upon FDA approval (no statistics reported).
Malik et al. (2020)	672, 57.0	Representative sample, survey platform	USA	May	PP	Exact wording n.a. If a COVID-19 vaccine were available and recommended for participants, would they accept it: Strongly disagree/ Disagree/ Neutral/ agree/ Strongly agree (5 points)	10	YR	n.s. OR (female vs. male): 0.51–1.02 *p* = 0.07
McAndrew and Allington (2020)	1,663, 51.4 GBR 1,198, 51.3 USA	Nationally representative samples, online panels	GBR USA	Jun	PP	‘When a Coronavirus (COVID-19) vaccine becomes available, do you think you will or will not get vaccinated?’ Definitely will get vaccinated/ Probably will get vaccinated/ Probably will not get vaccinated/ Definitely will not get vaccinated (4 points)	12	NA	UNSURE UK sample: gender no significant association with COVID-19 vaccine intentions in ordinal logistic regression model results *p* = 0.908/0.883 US sample: female respondents more vaccine-hesitant than men *p* = 0.005/0.011
Murphy et al. (2020)	1,041, 51.5 IRL 2025, 51.7 GBR	Nationally representative samples, e-mail from survey company	IRL GBR	Mar–Apr	PP	‘If a new vaccine were to be developed that could prevent COVID-19, would you accept it for yourself?’ Yes/ No/ Maybe	12	NA	Yes Both samples: those vaccine hesitant were more likely to be female (OR = 1.62 [1.18–2.22] IRL/ 1.43 GBR [1.14–1.80]) but not those vaccine resistant (OR = 1.24; [0.77–2.00] IRL OR = 1.05 [0.69–1.60] GBR).
Nzaji et al. (2020)	613, 49.1	Congolese Health Care Workers, recruited in several hospitals	COD	Mar– Apr	PR	‘If a COVID-19 vaccine was available, I would have it.’ Yes/ No	7	YR YN	Yes Logistic regression model: OR (male vs. female) = 1.17 [1.15–2.60] p = 0.008
Neumann-Böhme et al. (2020)	7662, NA	Representative samples	DNK FRA DEU ITA PRT NLD GBR	Apr	PR	Exact wording n.a. Willingness to get vaccinated against COVID-19 if a vaccine would be available: Willing/ Not sure/ Not wanting to get vaccinated	5^g^	NA	Yes Significantly higher proportion of men were willing to get vaccinated (77.94% vs. 70.15%, *p* < .001)
Olagoke et al. (2020)	501, 55.29	Sampling via crowdsourcing platform	USA	Mar	PP	‘If there is a preventive vaccine against COVID-19, how likely are you receive the vaccine?’ 1 (extremely unlikely) – 5 (extremely likely)	9	YR YN	n.s. *p* = 0.0948
Papagiannis et al. (2020)	461, 74.0	Convenient sample of health care workers, personal interview in hospitals	GRC	Feb	PR	‘Will you be vaccinated for SARS-CoV-2?’ Yes/ Uncertain/ No	7	YR	Yes There was a significant difference in gender concerning willingness to be vaccinated against SARS-CoV-2 with more male health care workers reporting that they would be vaccinated for COVID-19 than females (58.5% vs. 39%, respectively, p = 0.001).
Paul et al. (2020)	32,361, 74.9	Well-stratified non-representative sample, panel study, networks + mailing lists	GBR	Sept–Oct	PP	‘How likely to do you think you are to get a COVID-19 vaccine when one is approved?’ 1 (very unlikely) – 6 (very likely)	9	YR YN	Yes Groups at increased risk for uncertainty and unwillingness to vaccinate against COVID-19 were women (uncertain: RRR =1·45; 95% CI: 1·27 to 1·65; unwilling: RRR = 1·52; 95% CI: 1·24 to 1·86)
Perlis et al. (2020)	19,058, 53.3	Representative sample, sampling platform	USA	Jul	No PR	‘If a vaccine against COVID-19 was available to you, how likely would you be to get vaccinated?’ Extremely likely/ Somewhat likely/ Neither likely nor unlikely/ Somewhat unlikely/ Extremely unlikely	10	YR YN	Yes Women (62%) were less likely to say they would pursue vaccination than men (71%).
Pogue et al. (2020)	316, 49.38	Representative sample of census data, e-mail notification through survey panel	USA	Sept	PR	‘I am likely to be vaccinated for COVID-19 when a vaccine becomes available.’ strongly agree, somewhat agree, neither agree nor disagree, somewhat disagree, strongly disagree (5 points)	10	YR YN	n.s.
Prati (2020)	624, 54.0	Snowball sampling, social media	ITA	Apr	PR	‘Assume that your local health authority makes freely available a vac-cine against SARS-CoV-2. Do you intend to get the vaccine?’ Yes/ Do not know/ No	8	NA	n.s. Gender did not have an influence on intention to receive the vaccine Yes vs. No OR (women): 1.04 [0.51–2.13] Do not know vs. Yes OR (women): 1.15 [0.65–2.03]
Qiao et al. (2020)	1,062, 79.8	College student sample of one college in South Carolina, e-mail invitation	USA	Sept–Oct	PP	‘How likely will you get a COVID-19 vaccine when it is available?’ 1 (definitely not take it) – 5 (definitely take it)	6	NA	Yes Male college students report higher levels of COVID-19 vaccine acceptance in hierarchical linear regression (*p* = .03).
Reiter et al. (2020)	2,006, 56.0	Convenience sample, online survey panel	USA	May	PR	‘How willing would you be to get the COVID-19 vaccine if it was free or covered by health insurance?’ Definitely not willing/ Probably not willing/ Not sure/ Definitely willing/ Probably willing	8	YR YN	Yes Participants were less likely to be willing to get a COVID-19 vaccine if they were female. Multivariable correlates: RR = 0.91, CI: 0.87–0.96 Bivariate correlates RR: RR [female vs. male]: 0.85 (0.80–0.90)
Rhodes et al. (2020)	2,018, 49.7	Nationally representative sample of Autralian parents, part of a poll	AUS	Jun	PR	Exact wording n.a. Accept COVID-19 vaccine: Yes/ Not sure/ No	8	YR YN	Yes OR (female vs. male): 0.63 [0.50–0.80] *p* < 0.001
Roozenbeek et al. (2020)	700, NA 700, NA 700, NA 1,050 + 1,150 *700, NA*	Representative samples for age and gender, market research company/panel provider	USA ESP MEX GBR^e^ *IRL*^*f*^	Apr–May	PR	Whether participant would get vaccinated against COVID-19 if a vaccine were to become available: Yes/ No	9	YR YN	Yes Being male is associated with an increased likelihood to get vaccinated against COVID-19.
Salali and Uysal (2020)	1,088, NA 3,936, NA	Snowball sample, social media	GBR TUR	May	PR	‘If a new vaccine for COVID-19 is developed, would you get yourself and, if you have any, your children vaccinated?’ Yes/ Not sure/ No	6^g^	YR	UNSURE Men in Turkey more likely to accept a COVID-19 vaccine TUR: OR 1.47 [1.26–1.71] GBR: OR 1.44 [0.99–2.1]
Sethi et al. (2020)	4,884, 69.9	Convenience sample, social media networks, national radio, news articles, Clinical Research Network website and newsletter, text messaging service of general practices	GBR	Sept–Oct	PP	Exact wording n.a. Approved COVID-19 vaccine: Interested/ Unsure/ Not interested	3	YR YN	n.s. Males (OR = 3·47) and females (OR = 3·27) were both equally likely to take the approved vaccine.
Sherman et al. (2020)	1,494, 51.0	Representative of general population, research panel	GBR	Jul	PR	‘When a coronavirus vaccination becomes available to you, how likely are you to take it?’ 0 (extremely unlikely) – 10 (extremely likely)	12*	YR YN	n.s. *p* = .366
Taylor et al. (2020)	3,674, 43.0	Representative sample, survey sampling company	USA CAN	May	PR	‘If a vaccine for COVID-19 was available, would you get vaccinated?’ Yes/ No	9	NA	UNSURE Significant but according to sample size and Cohen trivial correlation between female gender and vaccination refusal *r* = .10, *p* < 0.001
Thaker (2020)	1,040, 58.6	Nationally representative sample, online panel	NZL	Jul	PP	‘I intend to get vaccinated against the coronavirus.’ 1 (strongly agree) – 5 (strongly disagree) with 3 = neither agree nor disagree	10	YR YN	Yes *p* (of negative standardised Beta for female) < .05
Thorneloe et al. (2020)	1,149, 63.2	Non-representative sample, social media, emails, research company	GBR	Apr–Jun	PP	‘If a vaccine was available for COVID-19, I would want to receive it.’ Strongly disagree/ Disagree/ Neither agree nor disagree/ Agree/ Strongly agree (5 points)	7	YR	n.s. Univariable analysis: OR: 0.93 [0.76–1.14], *p* = 0.487 Multivariable analysis: OR: 0.93 [0.72–1.21], *p* = 0.600
Unroe et al. (2020)	8,243, 87.2*	Representative sample for nursing home staff in Indiana, text message	USA	Nov	PR	‘If a vaccine is approved for use by the FDA for COVID-19, will you be willing to get it as soon as it is available?’ Yes/ No	8	YR YN	Yes Male staff more willing to receive the vaccine p < .0001
Vai et al. (2020)	2,223, 69.6	Convenience sample, advertisement by authors, universities, city social groups, social media	ITA	Feb–Mar	PR	Exact wording n.a.	6	YR YN	n.s.
Wang et al. (2020b)	2,058, 54.2	Stratified random sample representative for age and location, online survey platform	CHN	Mar	PR	‘If a COVID-19 vaccine is successfully developed and approved for listing in the future, would you accept vaccination?’ Yes/ No	9	YR YN	Yes Among those who would accept vaccination, male (OR = 1.25, 95% CI: 1.03–1.52), respondents were more likely to accept COVID-19 vaccination as soon as possible *p* = 0.03
Wang et al. (2020a)	806, 87.5	Nurses, e-mail	HKG	Feb–Mar	PR	Exact wording n.a. Whether intended to accept COVID-19 vaccination when it is available: Intend to accept/ Not intend to accept/ Undecided’	7	YR YN	Yes In the multiple multinomial regression, […], male (adjusted odds ratio (OR): 2.78, 95% confidence interval (95% CI): 1.69–4.58) were more likely to have intentions to accept COVID-19 vaccination […]
Ward et al. (2020)	5,018, 52.4	Representative population sample, online research panel/firm	FRA	Apr	PP	Exact wording n.a. Respondents were asked whether they would agree to get vaccinated if a vaccine against the COVID-19 was available: Certainly/ Probably/ Probably not/ Certainly not.	11	YR YN	Yes Women were more likely to refuse the vaccine. OR vaccine refusal (men vs. women): 0.75 [0.65; 0.86] Against vaccination in general vs. acceptance: OR (male vs. female): 0.56 [0.44; 0.72], *p* < .001
Williams et al. (2020)	527, 57.0	Convenience sample of participants 65 and older or chronic respiratory disease, sample recruited from previous projects	GBR	Apr	PR	‘If a vaccine for coronavirus becomes available, would you want to receive it?’ I definitely would not want to receive it/ I probably would not want to receive it/ Unsure/ I probably would want to receive it/ I definitely would want to receive it (5 point)	8	YR YN	n.s. There were no differences between males and females, t(1, 523) = 1.45,*p* = .14.
Wong et al. (2020)	1,159, 66.0	Snowball sample, social media	MYS	Apr	PR	‘If a vaccine against COVID-19 infection is available in the market, would you take it? Definitely not/ Probably not/ Yes possibly/ Yes probably/ Yes definitely (5 points)	7	YR YN	Yes […] males have greater odds of a definite intention to take the COVID-19 vaccine (OR = 1.44, 95% CI 1.11–1.87) than do females. *p* < .01.

Reported Gender Differences are displayed as in the respective papers. PP, Preprint; PR, Peer-reviewed; YR, frequencies for yes vs. rest categories reported; YN frequencies for yes vs. no categories reported. *weighted data according to population characteristics. ^a^ISO 3166 Alpha-3 country code. ^b^other Arab countries, Asian countries, EUE, NNN, SRR, AUS, NZL. ^c^response categories reported differently in method and result section (6 vs. 5 points) ^d^DEU, IND, ITA, MEX, NGA, POL, RUS, ZAF, South KOR, SGP, ESP, SWE, GBR, USA. ^e^for the first UK sample, vaccination intention was not assessed. ^f^for Ireland, vaccination intention was not assessed. ^g^gender proportion was not displayed

## Results

### Description of the studies

Sample sizes, sampling techniques, countries and month of assessment, publication type, item wording for the variable of interest, as well as quality ratings and reported gender differences can be seen in Table 1. Sample sizes ranged from 128 (Grech et al. 2020a) to 32,361 (Paul et al. 2020) participants with a total of 195,974 people across all 60 studies. The vast majority (70%) of studies, namely 42, had sample sizes of over 1,000 participants. Most papers (*n* = 35) were peer reviewed, but a substantial number were preprints (*n* = 24), and one was a report of scientific surveys made accessible online (Perlis et al. 2020). Surveys took place in 40 different countries. Most papers included samples from the USA (*n* = 22), UK (*n* = 13), Italy (*n* = 5), France (*n* = 5) and Australia (*n* = 4). Twenty-three studies took place in Europe exclusively.

Wording of the vaccination intention item was similar across the surveys. Most items asked about ‘likelihood’, ‘intention’ or ‘willingness’ to vaccinate or ‘acceptance’ of a COVID-19 vaccine. However, response categories varied from two (‘yes’, ‘no’) to five or more categories and one with 11 categories (Sherman et al. 2020). Many studies (*n* = 24) explicitly included a ‘not sure’/‘undecided’/‘maybe’-response category. We included studies that were conducted from February 2020 (Papagiannis et al. 2020) to November 2020 (Barry et al. 2020). Most studies (*n* = 15) took place in April 2020 (including those studies lasting more than one month), few studies were conducted in February and November (*n* = 2 each). Three studies did not report the time or period of recruitment (Pogue et al. 2020; Al-Mohaithef and Padhi 2020; Lucia et al. 2020). Results of a quality appraisal for the 60 studies are reported in the Supplementary Material.

### Gender differences in vaccination intentions

Thirty-six studies report significant gender differences in vaccination intentions in their result section for the whole sample. Male gender was associated with a greater likelihood of intending to accept a COVID-19 vaccine in 35 studies (58%). Only one study (Lazarus et al. 2020), reported men to be less likely to intend to accept of the vaccination compared with women. In five studies (Butter et al. 2020; Davis et al. 2020; McAndrew and Allington 2020; Salali and Uysal 2020; Khubchandani et al. 2021) results were not clear because significant gender differences could be found only in some subgroups and analyses but not in others. Most studies recruited only from the general adult population (*n* = 41). Twelve looked exclusively at health care workers and/or health care students (Grech et al. 2020a; Papagiannis et al. 2020; Barry et al. 2020; Lucia et al. 2020; Gadoth et al. 2020; Grech and Gauci 2020; Grech et al. 2020b; Nzaji et al. 2020; Kose et al. 2020; Kwok et al. 2020; Unroe et al. 2020; Wang et al. 2020a). Of those, eight reported significant gender differences (66.7%) as can be seen in Table 1. Four studies purposefully oversampled HCWs or key workers (Butter et al. 2020; Detoc et al. 2020; Dror et al. 2020; Grüner and Krüger 2020) to compare their intentions with the general population. Of those, only Butter et al. (2020) analysed gender differences separately for the two groups. They reported a significant association of COVID-19 vaccine hesitancy and being female *only* for key workers (mainly individuals employed in positions in health care, education and childcare or positions crucial for providing food, necessities and utilities).

### Meta-analytic results

Forty-six papers (77%) included frequency statistics for the calculation of averaged odds ratios (ORs) or they were provided by the authors upon request. This is noted in Table 1 in the column Frequencies. Roozenbeek et al. (2020) provided us with data from more countries and months than in the original paper which is why we have a larger sample for our own calculations then they did in their paper.^2^ For Sethi et al. (2020), we computed the frequencies for the ‘yes’-category from the frequencies of the other categories given in the paper. Loomba et al. (2020) conducted their study in the USA and UK but only data for the UK was available for meta-analytic computations. Daly and Robinson (2020) had frequencies for their assessment in April and October. We used data for April after verifying that follow up data for October did not make a big difference for our calculations. We conducted meta-analytic computation of the available data.

Data were available for 141,550 female and male participants, excluding people not identifying as male or female or with missing data. Of those papers not providing frequency statistics (*n* = 14), seven papers (50.0%) reported significant gender effects in their results section in favour of men and two papers each found significant effects for one of two subgroups. For the papers with reported or provided frequency statistics this percentage was higher with 60.9% of the papers (*n* = 28) reporting significant gender effects in favour of men. Mean quality rating of the papers without frequencies was *M* = 7.92 (*SD* = 2.3) with six papers (42.9%) with a rating of nine and higher (up to 12 which was the maximum). Mean quality rating of the papers with frequency statistics provided was *M =* 8.02 (*SD* = 2.19) with 18 papers (39%) with a rating of nine and higher and three papers with a rating of 12. Mean quality ratings did not differ, *U* = 294.00, *Z =* −0.495*, p* = 0.62.

**Fig. 2.**
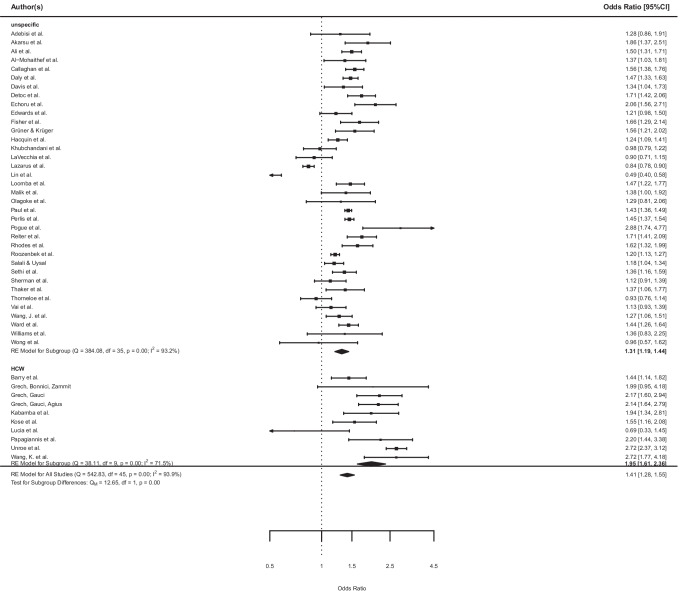
Forest plot of the odds of men reporting the intention to get vaccinated against COVID-19 compared to the reference group of women in unspecified samples (above) and Health Care Workers Samples (HCW, below). Results are expressed as odds ratio (OR) and 95% confidence intervals

Not all of the 46 papers had frequencies broken up into every answer category but summarized over several categories so that absolute ‘no’-answers could not be obtained for all of them. We therefore compared ‘yes’-answers with the rest-categories, that is, all but the ‘yes’-categories, including ‘no’ and ‘not sure’-answers. Wang et al. (2020b) was the only study that contained ‘delay of vaccination’ in the rest-category. The averaged OR was 1.41, 95% CI [1.28, 1.55] with higher odds for men than for women. This effect was significant, *z* = 7.10, *p* < .0001. The heterogeneity among the studies was substantial with *I*^2^ = 93.87%, *Q *= 542.83, *p* < 0.0001. The lowest OR was 0.49, 95% CI [0.40, 0.58] and the highest OR was 2.88, 95% CI [1.74, 4.77]. Figure 2 displays the corresponding forest plot.

Moderator analyses with study quality, first month of assessment and a HCW status as moderators revealed a significant moderation effect, *F *= 5.22, *p* = 0.004 (Ritchie et al. 2021; Grüner and Krüger 2020). Model results showed that only the factor HCW was a significant moderator for the observed study effects, *t* = 3.51, *p* = 0.001 (quality: *t* = −0.36, *p* = 0.720; month: *t* = 0.03, *p* = 0.975). The amount of heterogeneity *R*^*2*^ accounted for was 25.26%. Subgroup analysis revealed a significant subgroup difference for the yes vs. rest analysis with *Q*_*M*_ = 23.65, *p* = 0.00. Heterogeneity in the HCW subsample was lower than in the other subgroup but substantial in both (see Fig. 2). Averaged odd ratios for the subgroup of HCW was OR 1.79, 95% CI [1.61, 2.36] vs. OR 1.31, 95% CI [1.19, 1.44] for the unspecific sample.

## Discussion

In our systematic review we investigated gender differences in COVID-19 vaccination intentions. In our meta-analysis of averaged odds ratios across all the studies that provided us with the necessary frequency data (*n* = 46) we found an overall significant gender difference with males being on average 41% more likely to report that they intended to receive a vaccine (rather than being unwilling or undecided) compared with women. Quality ratings of the studies or first month of assessment did not have a significant impact on study effects. Subgroup analyses in response to our moderator analyses revealed that gender effects were even higher among health care workers (HCWs) compared with unspecific samples. However, this result must be interpreted cautiously because in HCW samples gender proportions were highly unbalanced and the number of studies with HCW samples was comparatively small.

Our finding that men showed on average a higher COVID-19 vaccination intention supports initial trends indicating systematic gender differences in reviews of COVID-19 vaccination intention (Galanis et al. 2020; Lin et al. 2021; Robinson et al. 2020). They are also in line with research on other vaccinations. For example, a study of vaccination coverage among adolescents found that females had a lower likelihood of being fully vaccinated compared with men (Sakou et al. 2011). Men have also been found to have higher vaccination rates than women in the case of influenza and pandemic influenza vaccinations (Bish et al. 2011; Pulcini et al. 2013; Jiménez-García et al. 2010).

### Vaccination intentions and actual vaccination uptake

In our efforts to compare COVID-19 vaccination intentions with the uptake of COVID-19 vaccinations, the majority of data has not yet been broken down by gender. In the COVID-19 Sex-Disaggregated Data Tracker (Global Health 50/50 2021), data only refer to the proportion of men/women in a country among all vaccinated people. This is skewed given that in some countries small numbers of people have been offered the vaccine to date.

There is much less data on the proportion of men and women who have accepted an offer to be vaccinated. In Germany, a representative survey conducted in August 2021 with 4,144 adults, showed that 79% of men and 73% of women reported that they have received a first vaccination dose (Huebner and Wagner 2021). In Austria, as of October 10, among most age groups (55 to over 84 years old), more men than women received a first dose of the COVID-19 vaccine (e.g. 98% of men vs. 90% of women among those aged over 84) (Bundesministerium Soziales Gesundheit Pflege und Konsumentenschutz Österreich 2021). Only in two age groups, namely between 15 and 24 years and 45 to 54 years, slightly more women had been vaccinated by mid-October. In the UK, overall 90.1% of females compared to 87.7% of males have been vaccinated with at least one dose since the vaccinations started (National Health Service 2021).

Evidence about vaccination uptake among HCWs in the UK and USA support our findings about female HCWs being more hesitant to get vaccinated. In the SIREN study in the UK on 29,378 hospital personnel, male HCWs were significantly more likely to be vaccinated than female HCWs, namely 90.8% of men vs. 88.1% of women (Hall et al. 2021). Among members of the Athens Medical Associations, more men (86.4%) than women (83.8%) were vaccinated. This difference failed to reach significance though. In the USA by July 2021, in a representative sample of 1,591 HCWs, female HCWs were less likely to be vaccinated, with 69% of female HCWs compared with 79% of male HCWs being vaccinated (Lazer et al. 2021).

Many of the studies included in this review asked individuals about their intentions to get the vaccine before a vaccine was available. It is well established that intentions do not always materialise into behaviour (Sheeran and Webb 2016). Usually, people are more likely to state they intend to do something and subsequently fail than the other way around. For example, in the field of physical activity, people often intend to exercise but do not always successfully translate this intention (inclined abstainers) into behaviour (Rhodes and de Bruijn 2013). In contrast, COVID-19 vaccine uptake in the UK is currently higher than anticipated, e.g. 64% of UK adults intended to get the vaccine when surveyed in September/October 2020 (Paul et al. 2020), while over 78.5% of people have received the first dose of the vaccine one year later (Government UK 2021b).

### Individual vs. policy factors

The fact that vaccination uptake was in most cases higher than indicated by early surveys may be attributable to a number of factors. Thinking of the intention-behaviour gap, a certain proportion of women (but also men) who had expressed low intentions, turned out to be ‘disinclined actors’, i.e. people who originally did not intend but nevertheless acted on something (Sheeran 2001). Information campaigns and the implementation of roll out may have addressed individual modifiable barriers that underpinned vaccine hesitancy at the time the surveys were conducted. In this respect, it can be considered a success that early data helped policy makers increase uptake and diminish gender disparities. In part, the on average lower intentions stated by women found in our meta-analysis may have been overcome by several factors (especially in high-income countries). Rising infections and the associated increased mortality, positive experiences with the COVID-19 vaccination by millions of people and very high initial uptake among high-risk groups positively influencing perceived norms to accept the vaccination may have contributed to this. Another important influence would have been policies around vaccination passports and increased personal freedom.

Regarding current vaccination mandates and policies, no country has a federal vaccination mandate. However, in the USA, many institutions, including universities, hospitals and big companies such as Walmart, require a vaccination by their employees or are about to install a vaccination mandate (Hals 2021). Additionally, the new Biden–Harris Administration will demand vaccination requirements for staff within all Medicare and Medicaid-certified facilities (Centers for Medicare and Medicaid Services 2021). In the UK, by 11 November 2021, care home staff must be fully vaccinated (Government UK 2021a). In Austria, similar to the USA, certain institutions in certain regions are allowed to and do require a COVID-19 vaccination, especially for new staff members (Tempfer 2021). In Germany, legislation to make COVID-19 vaccinations mandatory for health care workers has recently been passend and will come into effect in March 2022 (Bundesgesundheitsministerium 2021). A vaccine mandate for the general German population is also being discussed. These differences in actual vaccination policies and next to that different ways of promoting the vaccines and communicating vaccination information may play an important role in convincing vaccine non-intenders to get vaccinated.

## Limitations

Some limitations have to be addressed. We were not able to compare vaccination intentions in men and women among subgroups, for example, age groups or education levels. Therefore, we cannot rule out that our findings may be more or less pronounced in certain subgroups. We used a dichotomous format in our analyses; therefore, we were not able to see if women were maybe more hesitant but not strongly rejecting of the vaccine. Additional analyses distinguishing between people answering ‘yes’ or ‘no’ to vaccine acceptance with certainty and those being ‘unsure’ revealed that a greater proportion of women reported being ‘unsure’. Respondents being unsure might have been more easily convinced to take the vaccine once the campaigns started in comparison with those having had a strong negative opinion. In addition, meta-analytic calculations have not been adjusted for potential confounders such as country or study design. Accordingly, comparability between studies included in the meta-analysis is limited which is reflected in a rather high heterogeneity score.

Our search was conducted from November 2020 to January 2021 and therefore our findings do not incorporate if vaccination intentions changed from then on.

## Implications for policy and practice

Even if a large fraction of people – at least in high-income countries – is vaccinated already, the number of those who are not remains high. In the USA by the end of October 2021, 44% of the population were not fully vaccinated, in the UK 33% and in Germany 34% (Ritchie et al. 2021). In most countries where availability of vaccines is sufficient, people who had a high intention should be vaccinated by now. It might be necessary to focus on policy measures rather than individual psychological factors to reach the last share of unvaccinated people. It would be interesting to compare the policy measures in their effectiveness to convince still unvaccinated people in different countries. In the field of HCW, the next step is to find out how to convince hesitant women (and men) to get the vaccine.

## Supplementary Information

ESM 1 (DOC 67 kb)

## Data Availability

The data that support the findings of this study are available from the corresponding author upon reasonable request.

## Footnotes

1 Sixteen papers were only excluded from meta-analytic calculations due to the authors not providing us with the necessary data. Three papers were completely excluded. One was a preprint-version of a published article we already included, another one reported an outcome item for vaccination intention that included questions about the assumptions that the COVID-19 vaccination would be a good way to protect oneself and therefore differed too widely from the usually used vaccination intention item. The last one was a preliminary report of a study not properly analysed yet (and only reported gender differences for a specific age group).

2 Additional frequencies were provided for: Australia, China, Germany, Spain, France, Italy, Japan, South Korea, Mexico, Sweden, the UK and USA.
