# Supplementary material for: Gender differences in the intention to get vaccinated against COVID-19: a systematic review and meta-analysis
Source: Z Gesundh Wiss. 2022 Jan 7:1–25. Online ahead of print. doi: 10.1007/s10389-021-01677-w (PMC8739532; doi:10.1007/s10389-021-01677-w)
Supplement: Supplementary file 1 — (DOC 67 kb) [file 10389_2021_1677_MOESM1_ESM.doc]

**Supplementary Material**

**quality Appraisal**

**Methods**

We opted for nine criteria that were rated on a 3-point scale (see Table S1). The degree to which the study samples mirrored the general population, gender balance, the description and specification of the study population, the participation rate and the length of the recruitment period were evaluated. Justification of sample size, definition of outcome variables, treatment of missing data as well as the disclosure of statistical methods were also looked at.

Ratings for each criterion, except for criteria four, six and eight (due to limited suitability to all of the studies) were summarized into one score. Regarding the gender proportion- and time recruitment-criteria, some papers did not hold the needed information. We decided to code this with zero It is a limitation we found necessary to account for since these are important factors in the context of our research.

**Results**

The sum scores computed with six from nine criteria ranged from 0 to 12. In our rating, the lowest sum score awarded was three for two studies (Al-Mohaithef and Padhi 2020; Sethi et al. 2020) and the highest rating was 12 given to five papers (Sherman et al. 2021; Murphy et al. 2020; Fisher et al. 2020; McAndrew and Allington 2020; Lazarus et al. 2020). Mean quality rating for all studies was *M* = 8.0 (*SD* = 2.19). Fourteen studies were assigned 50% or less of the possible credits (Sethi et al. 2020; Salali and Uysal 2020; Qiao et al. 2020; Neumann-Böhme et al. 2020; Lucia et al. 2020; Kwok et al. 2020b; Kose et al. 2021; Grüner and Krüger 2020; Gadoth et al. 2020; Echoru et al. 2020; Dror et al. 2020; Butter et al. 2020; Al-Mohaithef and Padhi 2020; Vai et al. 2020). Twenty-four studies had ratings of nine and higherSum Scores of quality ratings for each study can be seen in Table 2. Many papers (*n* = 24) assessed samples which were mostly or somewhat representative of the general population of the country or countries where the data were collected (Callaghan et al. 2020; Daly and Robinson 2020; Davis et al. 2020; Edwards et al. 2020; Fisher et al. 2020; Hacquin et al. 2020; La Vecchia et al. 2020; Lazarus et al. 2020; Loomba et al. 2020; Malik et al. 2020; McAndrew and Allington 2020; Murphy et al. 2020; Neumann-Böhme et al. 2020; Paul et al. 2020; Perlis et al. 2020; Pogue et al. 2020; Rhodes et al. 2020; Roozenbeek et al. 2020; Sherman et al. 2021; Taylor et al. 2020; Thaker 2020; Wang et al. 2020a; Ward et al. 2020; Guidry et al. 2021). Description of the samples and the recruitment can be seen in Table 2. Several studies had quite unequal gender distributions, with the proportion of one group accounting for 65% or more of the sample (*n* = 15) (Ali et al. 2020; Al-Mohaithef and Padhi 2020; Butter et al. 2020; Detoc et al. 2020; Echoru et al. 2020; Faasse and Newby 2020; Gadoth et al. 2020; Kwok et al. 2020b; Qiao et al. 2020; Papagiannis et al. 2020; Sethi et al. 2020; Unroe et al. 2020; Vai et al. 2020; Wang et al. 2020b; Wong et al. 2020).

Several studies had low quality ratings which were reflective of the reporting of the study findings rather than the study design (Neumann-Böhme et al., 2020; Perlis et al., 2020; Rhodes et al., 2020).

**Table S1**

**Quality criteria.**

| Criteria | Rating | | |
| --- | --- | --- | --- |
|  | 0 | 1 | 2 |
| 1 Representativeness of the general population of the country/countries | Selected groups/  Snowball sampling/Convenience sample | Somewhat representative | Truly representative |
| 2 Gender proportion | One group > 65% | One group  54 - 65% | Ca. 50% - 50% |
| 3 Study population clearly specified and defined (e.g. eligibility criteria) | No | Somewhat | Yes |
| 4 Participation rate of eligible participants | < 50% |  | At least 50% |
| 5 Recruitment of all participants in same time period | > One month | Up to one month | One week |
| 6 Sample size justification [power description, variance and effect estimates] | No |  | Yes |
| 7 Outcome measures clearly defined | Response category or variables not clear |  | Clear description of assessed variable and response categories |
| 8 Treatment of missing data explained | No |  | Yes |
| 9 Statistical methods described/appropriate:  What was used to determine statistical significance, description that enables repetition, assessment of statistical significance | No | Not all aspects fulfilled | Yes |

*Note.* Quality criteria used for quality assessment of studies. A sum score was computed including all quality criteria except for criterion four, six and eight.

**Additional Analyses**

Analysis: Meta-analytic calculations were done for averaged odds ratios comparing men and women answering “yes” or “probably yes” to the vaccination item in reference to those men and women answering “no” or “probably no”. In comparison to Figure 2 included in the paper, men and women giving an “Unsure”-answer (if available) were not considered for the analysis. Because we did not have frequencies for all answer categories for all of the studies but sometimes the “unsure” category was put together with the no-answers, the number of studies for this analysis is slightly lower than that for the analysis comparing yes answers with the rest of the answers (40 studies vs. 46 studies). In addition we separated for subgroups: Studies having an unspecific sample vs. studies having a health care worker sample.

Results: The overall averaged OR was 1.40 95% CI [1.26, 1.56] with men being more likely to have answered “yes” instead of “no” to the vaccination item than women. This effect was significant, *z* = 6.09, *p* < .001. The heterogeneity among the studies was substantial with *I*^2^ = 91.78%, *Q*(39) = 340.34, *p* < .001. The lowest OR was 0.49 95% CI [0.40, 0.58] and the highest OR was 3.03 95% CI [1.95, 4.70]. Moderator analyses with the moderators quality, first month of assessment and a categorial variable coding being a health care worker (HCW) or not, revealed an overall marginal significant moderation effect for the yes vs. no analysis *F*(3, 34) = 2.78, *p* = 0.056. Model results displayed a nonsignificant effect of the factors quality and month on study effects *p*’s > 0.6 but a significant effect for the factor HCW *t* = 2.59, *p* = 0.01. Subgroup analysis revealed a significant subgroup difference for the yes vs. no analysis Q_M_(1) = 5.88, *p* = 0.02. Heterogeneity in the HCW subsample was lower than in the other subgroup but substantial in both (see forestplot). Averaged odds ratios for the subgroup of HCW was OR 1.79 95% CI [1.33, 2.41] vs. OR 1.31 95% CI [1.18, 1.46] for unspecific samples in the yes vs. no analysis.

Figure S1.
